# Narrow band green light effects on headache, photophobia, sleep, and anxiety among migraine patients: an open-label study conducted online using daily headache diary

**DOI:** 10.3389/fneur.2023.1282236

**Published:** 2023-10-04

**Authors:** Richard B. Lipton, Agustin Melo-Carrillo, Mark Severs, Michael Reed, Sait Ashina, Timothy Houle, Rami Burstein

**Affiliations:** ^1^Department of Neurology, Albert Einstein College of Medicine, Bronx, NY, United States; ^2^Departments of Anesthesia, Critical Care and Pain Medicine, Beth Israel Deaconess Medical Center, Boston, MA, United States; ^3^Department of Anesthesia, Harvard Medical School, Boston, MA, United States; ^4^Allay, Baltimore, MD, United States; ^5^Vedanta Research, Chapel Hill, NC, United States; ^6^Departments of Neurology, Beth Israel Deaconess Medical Center, Boston, MA, United States; ^7^Massachusetts General Hospital, Boston, MA, United States

**Keywords:** trigeminovascular, pain, nociception, spinal trigeminal nucleus, retina, visual cortex

## Abstract

**Background:**

Narrow band green light (NbGL) has been shown to relieve headache in small numbers of subjects but large-scale real-world assessments are lacking. The goal of this prospective, observational, open-label, real world study was to determine whether treatment with NbGL during the ictal phase of migraine, improves patients' perception of their headache, photophobia, anxiety and same-night sleep.

**Methods:**

The study was conducted in purchasers of the NbGL Lamp in two phases. In Phase I purchasers of the Lamp completed a survey and were asked to participate in a 6-week diary study. In Phase 2 participants completed daily diaries for 6 weeks. Specifically, they were asked to use their judgement/impression/perception when choosing between headache-improved or headache-unimproved after using the NbGL during acute attacks. Diary outcomes of interest included rates of attacks improve in responders (≥50%), non-responders (<50%), super-responders (≥75%), and super non-responders (<30%).

**Results:**

Of 3,875 purchasers of the Lamp for migraine, 698 (18%) agreed to participate, filled out a pre-study survey, and agreed to a 6-week daily headache diary. Complete data were provided by 181 (26%) participants. Using criteria above, 61, 39, 42, and 27% of participants were classified responder, non-responder, super-responder and super non-responder, respectively. Headache improved in 55% of all 3,232 attacks, in 82% of the 1,803 attacks treated by responders, and in 21% of the 1,429 attacks treated by non-responders. Photophobia improved in 53% of all attacks, 68% of the attacks in responders and in 35% of the attacks in non-responders. Anxiety improved in 34% of all attacks, 46% of the responders' attacks, and 18% of the non-responders' attacks. Sleep improved in 49% of all attacks, 59% of the responders' attacks, and 36% of the non-responders' attacks.

**Conclusion:**

This open-label real world study suggests that 2 h of treatment with the lamp during migraine attacks is associated with relief of pain and photophobia, reduction in anxiety, and improved sleep. The absence of rigorous diagnosis and a blinded contemporaneous control group limits the rigor of this interpretation. Improvement in photophobia, anxiety and sleep among the responders may be secondary to the improvement in the headache itself.

**Clinical trial registration:**

ClinicalTrial.gov (NCT04841083).

## Introduction

Nearly a decade ago, Noseda et al. reported that in blind migraine patients who could detect light, the headache is selectively exacerbated by blue light (1). In a subsequent series of pre-clinical and clinical studies they discovered a novel *retino-thalamo-cortical* pathway that links brain areas that process vision to brain circuits that mediate the classical pain of migraine, and to circuits involved in sensory as well as cognitive processing (thinking, memory, and attention) (1–3). They subsequently observed that in migraine patients with normal eyesight, the headache is exacerbated by narrow bands of light that selectively activate short- or long-wavelength cones (i.e., photoreceptors activated by blue and red lights, respectively), while narrow band of green light (NbGL, 520 ± 10 nm) selectively activates medium wavelength cones and ameliorates headache (4). While studying the effects of different colors of light on migraine patients, they described patients who found blue and red lights extremely unpleasant even when their headache intensity did not increase with exposure. After studying these patients more carefully, they reported that during migraine, exposure to narrow bands of blue, amber and red lights can trigger/exacerbate feeling of stress, irritability, worry and anxiety. In contrast, NbGL not only ameliorated headache but also promoted feelings of calm and relaxation (5).

Subsequently, daily exposure to NbGL phototherapy was shown to reduced monthly headache days, as well as headache intensity and duration in chronic and episodic migraine patients (6). These human observational studies are supported by animal studies showing that exposure to NbGL attenuates responses of thalamic trigeminovascular neurons (4), and produces long-lasting antinociceptive effects, potentially by eliciting anti-inflammatory responses or modulating the endogenous opioid system (7–9). Based on this evidence, a therapeutic lamp that emits NbGL was developed as a treatment for migraine and as a strategy for reducing anxiety, for inducing feelings of calm and relaxation, and for promoting sleep.

The few clinical studies described above, included small numbers of patients and were conducted in the context of established patient-provider relationship. This context carries the potential for amplifying positive expectations based on trust, empathy, acceptance, genuineness, respect, and warmth, between patient and health care professional (10, 11). Real world evidence on the effects of NbGL on patients with migraine is here-to-for lacking. To close this gap while mitigating the potential influence of the provider-patient relationship, we designed a prospective, observational, open-label, real world study whose goal was to determine whether treatment with NbGL during the ictal phase of migraine, improves patients' perception of their headache, photophobia, anxiety and same-night sleep among US migraine patients (residing in 37 states) who purchased the NbGL-emitting lamp (Allay Lamp) and agreed to be enrolled in a real-world observational study.

## Methods

### Study overview

**Figure 1:**
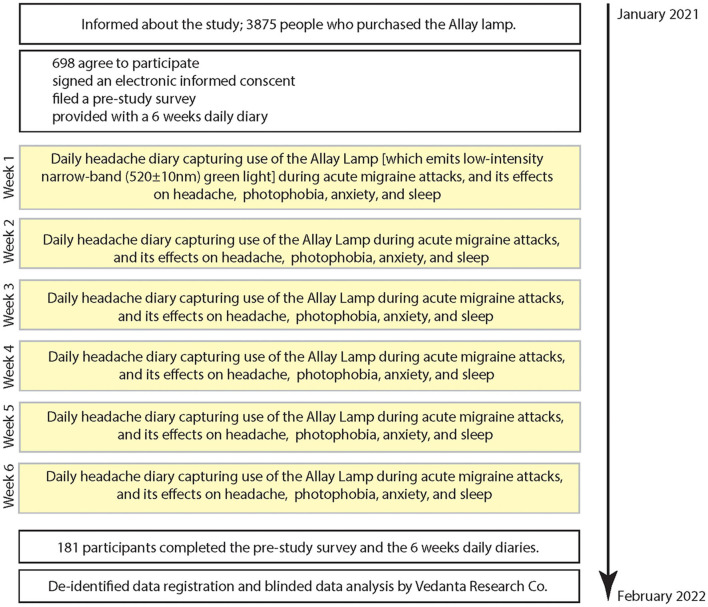
Study flowchart.

This study consists of 2 phases: (a) a pre-study survey capturing demographics and disease characteristics, and (b) a 6-weeks daily diary capturing occurrence of headache, use of Allay lamp (i.e., the intervention), and its effects on headache, photophobia, anxiety, and sleep (Figure 1). Data for the pre-study survey and the daily diaries were captured using a secure link to online website. To capture the effects of NbGL, participants were instructed to turn on the NbGL-emitting Allay Lamp only during an acute migraine attack, and only at a time in which they were able to spend at least 2 h in a room in which all other sources of light could be turned off (i.e., a room where the only source of light was the 520 ± 10 nm NbGL). After finishing their treatment with the Allay Lamp, they were asked to report duration of use, and their perception of the effects of treatment on their headache pain (i.e., did they think that NbGL improve their headache. *note: in effort to not lead them, they were not asked to rate headache intensity, duration or functionality*), and on their photophobia experience (did treatment made light less aversive and eliminate the exacerbating effect of light on pain) and on anxiety (did treatment made them feel less anxious or more relaxed). They were also asked to note whether they thought that they slept better the night immediately following days they used the Allay Lamp. To capture as many attacks as possible, patients were instructed to repeat this protocol in every migraine attack in which they could spend the 2 h in an appropriate room. Primary and secondary outcomes were evaluated based on the daily diaries as specified in the Statistical analysis section.

### Ethics

The information and consent form, as well as the survey instrument, were reviewed by Ethical and Independent Review Services (Independence, MO, USA), which granted an exemption from the requirements of federal regulation 45 CFR 46.101(b) (2) and certified the exemption status of the Study (#20177) on December 3, 2020. Before initiating the survey, respondents read a description of the study objectives and requirements, confirmed that they understood the purpose of the study, and electronically consented to participate.

### Recruiting and inclusion criteria

People who purchased the NbGL-emitting Allay Lamp for their migraine were invited to participate in the study. Those who volunteered to participate, provided sociodemographic information and completed the AMS/AMPP migraine diagnostic module (12). The module captures the International Classification of Headache Disorders, 3rd edition (beta version, ICHD-3) criteria for migraine, including the following characteristics of head pain: unilateral location, throbbing, and moderate/severe intensity; the following associated symptoms: nausea, photophobia, and phonophobia; and exacerbation by routine activity (13). This self-report of symptoms has a sensitivity of 100% and specificity of 82% for episodic migraine diagnosis, and sensitivity of 91% and specificity of 80% for chronic migraine diagnosis (12). Responders meeting the ICHD-3 migraine criteria (based on the pre-study survey), were invited to participate in a 6-week in-home evaluation of potential benefits of the Allay Lamp. The incentive for participation was a $25 rebate on their purchase of the Allay Lamp.

### Intervention

Two-hours exposure to 520 ± 10 nm (peak ± range) NbGL delivered at low intensity (5–10 candela/m^2^) using the Allay Lamp. *We chose this narrow band of green light as it allows 90% of the light to be distributed within a range that is least likely to activate the blue and red sensing retinal cones and nearly maximally likely to activate the green sensing cones*. To reach these lighting conditions, participants were instructed to place the lamp outside their direct visual field (i.e., 4–6 feet behind or above a place where they were sitting or working) and dim it to a point at which they could see clear enough to be able to read effortlessly. They were instructed to use their regular abortive medications if they felt the need to do so after the 2 h in the green light (no record was collected on use of abortive medication at the end of each trial).

### Assessments

For this study, demographics included gender (male of female), age (years), height and weight which were used to calculate a body mass index. Headache characteristics included monthly headache days (MHDs), and the following migraine symptoms: laterality, throbbing, nausea, photophobia, phonophobia, osmophobia, neck pain and dizziness. MHD were calculated by asking how many days over the past 3 months participants were affected by migraine for all or part or the day. These data were used to classify people with migraine into three frequency denominated categories: low-frequency episodic, (LFEM, <8 MHD), high-frequency episodic (HFEM, 8–14 MHDs), and chronic migraine (CM, ≥15 MHDs,). The frequency of occurrence of each of the seven migraine symptoms was assessed using the following response options: Never, Rarely (<50% of the times or = to 50% of the time) or Always. Given the large variation in symptoms presentation of individual attacks (e.g., a dizziness could appear in one out of four attacks), we only used Always to assess the percentage of participants whose migraine attacks are associated with each of the seven symptoms (Table 1).

**Table 1:** Characteristics of participants.

		**All patients**	**Responders**	**Non responders**	***P*-value**	**Super responders**	**Super non-responders**	***P*-value**
Gender	Female	(163) 90%	(101) 92%	(62) 87%		(69) 90%	(41) 84%	
	Male	(18) 10%	(9) 8%	(9) 13%	0.446 (FE)	(8) 10%	(8) 16%	0.412 (FE)
Age		43.4 (13.1)	41.3 (12.1)	46.7 (14.1)	0.016 (MW)	40.9 (12.6)	48.08 (13.8)	0.006 (MW)
BMI		27.3 (7.3)	27.6 (7.7)	26.8 (6.6)	0.647 (MW)	27.4 (8.1)	26.8 (6.4)	0.978 (MW)
Headache Days		16.2 (9.2)	15.1 (8.9)	18.1 (9.4)	0.040 (MW)	14.1 (8.8)	19.7 (9.6)	0.002 (MW)
Headache intensity		6.4 (1.8)	6.5 (1.7)	6.1 (1.9)	0.199 (MW)	6.6 (1.6)	6.2 (1.9)	0.451 (MW)
Classification	LFEM	18%	23%	11%		26%	8%	
	HFEM	28%	27%	28%		26%	29%	
	CM	54%	50%	61%	0.13 (FE)	48%	63%	0.039 (FE)
1. Unilateral		45%	45%	48%	0.760 (FE)	27%	34%	0.275 (FE)
2. Throbbing		43%	44%	42%	0.879 (FE)	32%	34%	0.717 (FE)
3. Nausea		23%	25%	20%	0.471 (FE)	15%	13%	0.821 (FE)
4. Photophobia		52%	46%	62%	0.048 (FE)	30%	44%	0.029 (FE)
5. Phonophobia		46%	46%	48%	0.879 (FE)	30%	37%	0.278 (FE)
6. Osmophobia		34%	36%	32%	0.634 (FE)	24%	23%	1 (FE)
7. Neck pain		40%	38%	45%	0.439 (FE)	25%	34%	0.138 (FE)
8. Dizziness		22%	22%	23%	1 (FE)	14%	15%	0.821 (FE)

FE, Fisher Exact; MW, Mann-Whitney; LFEM, low frequency episodic migraine; HFEM, high frequency episodic migraine; CM, chronic migraine. Numbers in the bottom 8 rows depict percentage of participants reporting symptoms that ALWAYS accompany their migraine attacks.

### Definitions of responders, non-responders, super-responders, and super non-responders

Responders were those whose headache improved in ≥50% of attacks in which they used the Allay Lamp. Non-responders were those whose headache improved in < 50% of attacks in which they used the Allay Lamp. Super-responders were those whose headache improved in ≥75% of attacks in which they used the Allay Lamp. Super non-responders were those whose headache improved in <25% of attacks in which they used the Allay Lamp.

### Statistics

Proportions of female and male, LFEM, HFEM and CM in each group were compared using Fisher Exact Test. Age, BMI, MHD, and headache intensity values were first tested for normality using Shapiro-Wilk test. After determining distribution, a non-parametric, Mann Whitney *U*-test was used for comparisons between groups. Occurrence of associated symptoms (laterality, throbbing, nausea, photo-, phono- osmophobia, neck pain, dizziness) were analyzed and compared using Fisher Exact test. Incidences of improvement in headache, photophobia, anxiety, and sleep during attacks in which the NbGL was used, were summarized using descriptive statistics and frequency counts with percentages. Differences between groups were analyzed using Fisher Exact test. The number of hours patients in each group used the Allay Lamp in attacks in which the headache improved vs. attacks in which the headache did not improve were compared using the Mann Whitney test. Level of significance for all tests was set at 0.05. All analyses were done by an investigator who was blinded to the treatment outcome, group, tested parameter, or any of the demographic data.

As the study design allowed participants to treat multiple attacks and did not fix the number of attacks each participant could contribute to the analysis, it was also necessary to account for the potential clustering of outcomes within each participant. A generalized linear mixed-effects model was utilized that specified a random intercept for each participant. Additionally, because the number of attacks that each participant contributed to the analysis could associate with the outcome status (i.e., informative cluster size), we adjusted for the number of attacks per person as a covariate. Since the outcome was defined as treatment success, a binomial distribution and logit link were specified for the models. Descriptive models were then conducted to examine patterns in the treatment response using the same formulation as above but included age, sex, and baseline headache frequency as covariates. The analyses were conducted using R4.2 and R-Studio with the “lme4” package for the mixed models.

We also conducted generalized linear mixed effects models to examine the probability of successful treatment response within individuals over repeated attacks. These models utilized a binomial outcome to represent a successfully treated attack (i.e., no vs. yes) and accommodated the repeated attacks within individuals by specifying a random intercept for each participant. Because participants could treat as many attacks as they wished, there was a possibility that individuals who experienced a better response might treat more attacks (i.e., improved treatment outcome might lead to more green light exposures in a type of reverse causation). To consider this potential bias, two versions of the model were conducted. The first model treated the varying number of treated attacks as a random slope, thereby allowing the evaluation of between-participant heterogeneity in the trajectory of the probability of success over repeated attacks. The second model treated the attack number as a fixed effect, allowing direct estimation of the marginal probability of treatment success conditional on the attack number (i.e., 1 through N). The use of variance components and intraclass correlation (ICC) was estimated to examine the variation of attack success within and across individuals. The mixed models were all conducted using the “lme4” package in R 4.2.1 and R-Studio 2,023.03.0 + 386.

## Results

### Sample and demographics

There were 3,875 potentially eligible participants; those who had purchased the Allay Lamp for migraine. Of those, 698 (18%) people agreed to participate, filled out a pre-study survey, and agreed to complete a 6-week daily headache diary. Complete data were provided by 181 (26%) participants. Demographics are presented for all 181 participants. Of the 181 participants with complete data, 110 (61%) were responders to the Allay Lamp and 71 (39%) were non-responders (see definition in Methods). There were 77 (42%) super-responders, and 49 (27%) super non-responders (Table 1), leaving 55 (30%) participants with an intermediate response of 25 to <75%. The 181 participants were mostly women (90%), had a mean age of 43.4, represented both episodic (46%) and chronic (54%) groups, and the consistency of their migraine symptoms (i.e., symptoms accompanying each attack as defined by answers they provided in the pre-study survey) ranged between 20 and 50%. Of note, responders were 5 years younger, more likely to have episodic migraine, and experience photophobia less consistently than non-responders. Similarly, super-responders were 7 years younger, less likely to have chronic migraine, and experience photophobia less consistently than super non-responders.

### NbGL effects on headache

**Figure 2:**
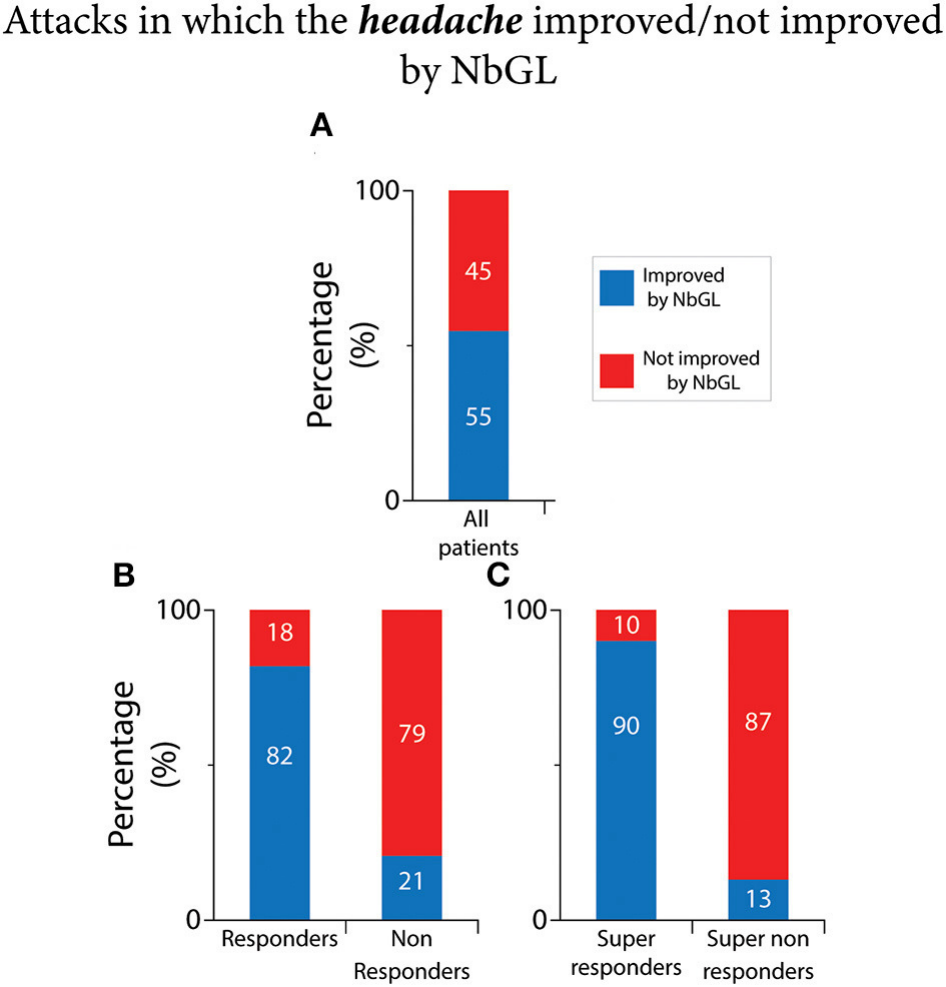
Narrow band green light (NbGL) effects on incidence of attacks in which the headache improved vs. not improved. **(A)** All patients. **(B)** Responders vs. non-responders. **(C)** Super-responders vs. super non-responders. The 100% (in **A–C**) represents incidences of headache improvement in all attacks in which the NbGL was used (all, 1,768/3,232; responders, 1,473/1,803; non-responders, 295/1,429; super-responders, 1,134/1,260; super non-responders, 135/1,035).

Of the 3,232 attacks recorded by the 181 participants [median 16 (9–25) attacks per person], exposure to NbGL improved the headache in 55% (1,768 attacks). Of the 1,803 headache attacks recorded by the 110 responders, 82% (1,473 attacks) improved by the exposure to NbGL (Figure 2, Table 2). In contrast, of the 1,429 headache attacks recorded by the 71 non-responders, only 21% (295 attacks) improved by the exposure to NbGL (*p* < 0.0001 DF = 1, Fisher exact) (Figure 2, Table 2). Of the 1,260 headache attacks recorded by the 77 super-responders, 90% (1,134 attacks) improved by the exposure to NbGL. In contrast, of the 1,035 headache attacks recorded by the 49 super non-responders, only 13% (135 attacks) improved by the exposure to NbGL (*p* < 0.0001 DF = 1, Fisher exact) (Figure 2, Table 2).

**Table 2:** Narrow band green light effects on headache, photophobia, anxiety, and sleep.

	**Participants**		**Attacks**		**Improved**							
					**Headache**		**Photophobia**		**Anxiety**		**Sleep**	
	**Count**	**Percent**	**Count**	**Percent**	**Count**	**Percent**	**Count**	**Percent**	**Count**	**Percent**	**Count**	**Percent**
Responders	110	61%	1,803	56%	1,473	82%	1,228	68%	832	46%	1,071	59%
Non responders	71	39%	1,429	44%	295	21%	497	35%	255	18%	517	36%
All patients	181	100%	3,232	100%	1,768	55%	1,725	53%	1,087	34%	1,588	49%

### Expected treatment success for a single attack

**Figure 3:**
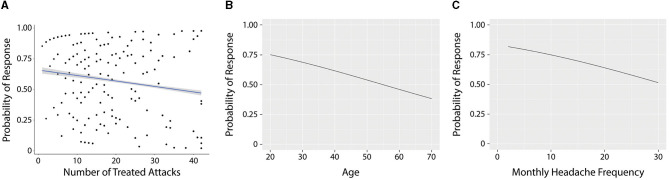
Association between the number of treated attacks and the probability of success **(A)**, age of participants and probability of success **(B)**, and frequency of attacks and probability of success **(C)**.

Across all participants, the expected treatment success for a single attack was 57.8% [(95% CI: 48.7–66.4%), *p* < 0.001, generalized linear mixed-effects model]. *This estimate is a conditional probability for a participant that treated 24.6 attacks*. Finding from the descriptive model identified the following patterns in treatment response: (a) the number of treated attacks across individuals was associated with a small reduction in treatment response probability, with individuals who treated more attacks reporting less success than those who treated fewer attacks (OR 0.97; 95% CI: 0.94–0.99), *p* < 0.020.) (Figure 3A); (b) older participants reported fewer successfully treated attacks, (OR 0.97; 95% CI: 0.95–0.99) (Figure 3B); (c) higher baseline headache frequency was associated with reduced treatment success (OR 0.95; 95% CI: 0.92–0.98) (Figure 3C). Interestingly, there was strong evidence for within-person clustering of treatment success, with an inter-class correlation coefficient (ICC) of 0.53, indicating that individuals tended to experience a similar probability of treatment success across attacks. That is, there is a treatment response group of individuals.

#### Sensitivity analyses-evaluating the threat of reverse causation

**Figure 4:**
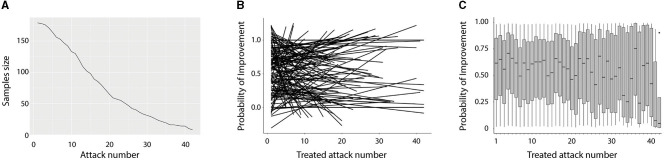
Sensitivity analyses evaluating the threat of reverse causation. **(A)** The sample size (*y*-axis) by the number of treated attacks (*x*-axis). *N* = 181 individuals treated at least one attack, with 50% treating 16 or more attacks, but only 25% of individuals treated 25 attacks. **(B)** A “spaghetti” plot that displays the probability of success using individual linear trajectories (*N* = 181) for each individual in the study across repeated attacks. Individuals varied considerably in their trajectories over time, with individuals experiencing increases, decreases, and stability in treatment response over attacks. **(C)** A box and whisker plot of the probability of treatment response (*y*-axis) by attack number (*x*-axis). The median (black line), 25th and 75th percentiles (gray box), and 95% CI are displayed across individuals who treated that number of attacks. Across the first 10 treated attacks, the mean probability of success ranged from 0.55 to 0.71.

During the study period, *N* = 181 participants treated 3,232 individual attacks. Participants treated a median (25th, 75th) of 16 (9, 25) (range: 1–42) attacks. Figure 4A displays the distribution of participants by attack number. At a crude level, the treatment success was 1,768/3,210 (52%) with *n* = 22 attack dispositions missing. Considering the clustering of response within individuals, the marginal likelihood (%) of treatment success was 64% (95% CI: 54–72%).

The potential for reverse causation due to previous successful treatment outcomes influencing a greater chance of future exposure to the treatment was evaluated. There was remarkable within-individual clustering, ICC = 0.67, suggesting that the probability of attack success was clustered within individuals (i.e., successful treatments tended to occur together within individuals). However, the mean trajectory of success did not meaningfully change across attacks, with each subsequent attack associated with only a small increase in the success probability, OR 1.01 (95% CI: 0.98–1.04), *p* = 0.506. Across individuals, there was considerable variability in initial treatment success and trajectory of success across attacks. Figure 4B displays this variability which is difficult to characterize as individuals experience a wide range of success probability and variable trajectory over repeated attacks. Figure 4C displays the probability of improvement across individuals for each treated attack. Although the probability of success diminished for the individuals who chose to treat >30 attacks, neither Figure 1 nor Figure 2 supports the notion that the marginal treatment estimate is primarily driven by reverse causality.

### NbGL effects on photophobia

**Figure 5:**
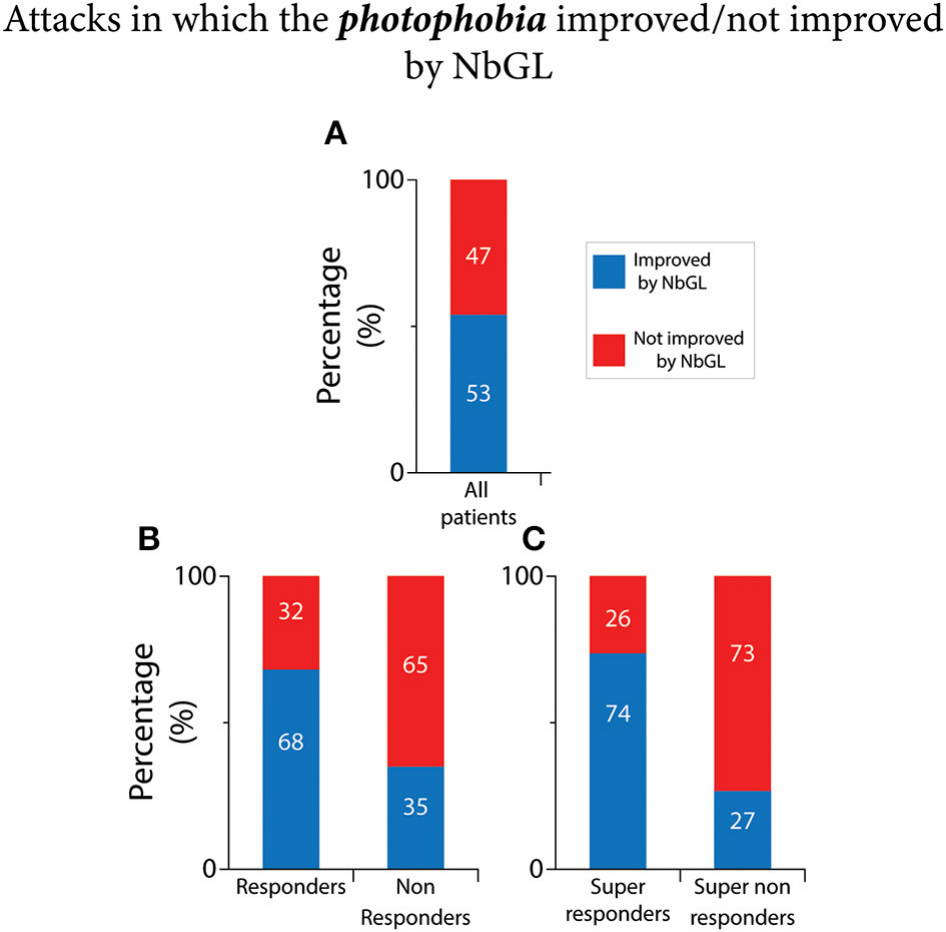
Narrow band green light effects on incidence of attacks in which the photophobia improved vs. not improved. **(A)** All patients. **(B)** Responders vs. non-responders. **(C)** Super-responders vs. super non-responders. The 100% (in **A–C**) represents incidences of headache improvement if all attacks in which the NbGL was used (all, 1,725/3,232; responders, 1,228/1,803; non-responders, 497/1,429; super-responders, 928/1,260; super non-responders, 275/1,035).

As shown in Figure 5 and Table 2, of the 3,232 recorded attacks, photophobia was improved in 53% (1,725 attacks) by the NbGL. In the 1,803 headache attacks recorded by the 110 responders, photophobia was improved in 68% (1,228 attacks) by the NbGL. In contrast, in the 1,429 headache attacks recorded by the 71 non-responders, photophobia was improved in only 35% (497 attacks) by the NbGL (*p* < 0.0001 DF = 1, Fisher exact). In the 1,260 headache attacks recorded by the 77 super-responders, photophobia was improved in 74% (928 attacks) by the NbGL. In contrast, in the 1,035 headache attacks recorded by the 49 super non-responders, photophobia was improved in only 27% (275 attacks) by the NbGL (*p* < 0.0001 DF = 1, Fisher exact).

### NbGL effects on anxiety

**Figure 6:**
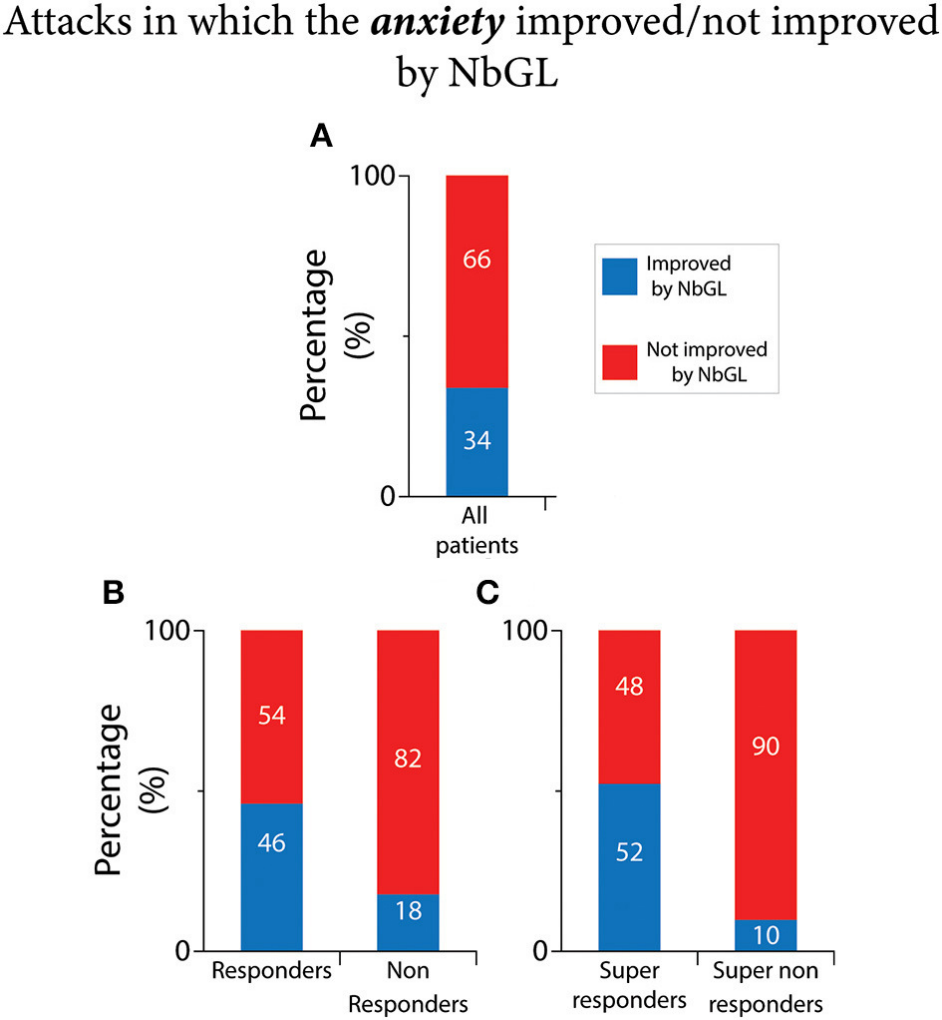
Narrow band green light (NbGL) effects on incidence of attacks in which anxiety improved. **(A)** All patients. **(B)** Responders vs. non-responders. **(C)** Super-responders vs. super non-responders. The 100% (in **A–C**) represents incidences of headache improvement if all attacks in which the NbGL was used (all, 1,087/3,232; responders, 832/1,803; non-responders, 255/1,429; super-responders, 660/1,260; super non-responders, 103/1,035).

As shown in Figure 6 and Table 2, of the 3,232 recorded attacks, anxiety was improved in 34% (1,087 attacks) by the NbGL. In the 1,803 headache attacks recorded by the 110 responders, anxiety was improved in 46% (832 attacks) by the exposure to NbGL. In contrast, In the 1,429 headache attacks recorded by the 71 non-responders, anxiety was improved in only 18% (255 attacks) by the exposure to NbGL (*p* < 0.0001 DF = 1, Fisher exact). In the 1,260 headache attacks recorded by the 77 super-responders, anxiety was improved in 52% (660 attacks) by the NbGL. In contrast, in the 1,035 headache attacks recorded by the 49 super non-responders, anxiety was improved in only 10% (103 attacks) by the NbGL (*p* < 0.0001 DF = 1, Fisher exact).

### NbGl effects on sleep

**Figure 7:**
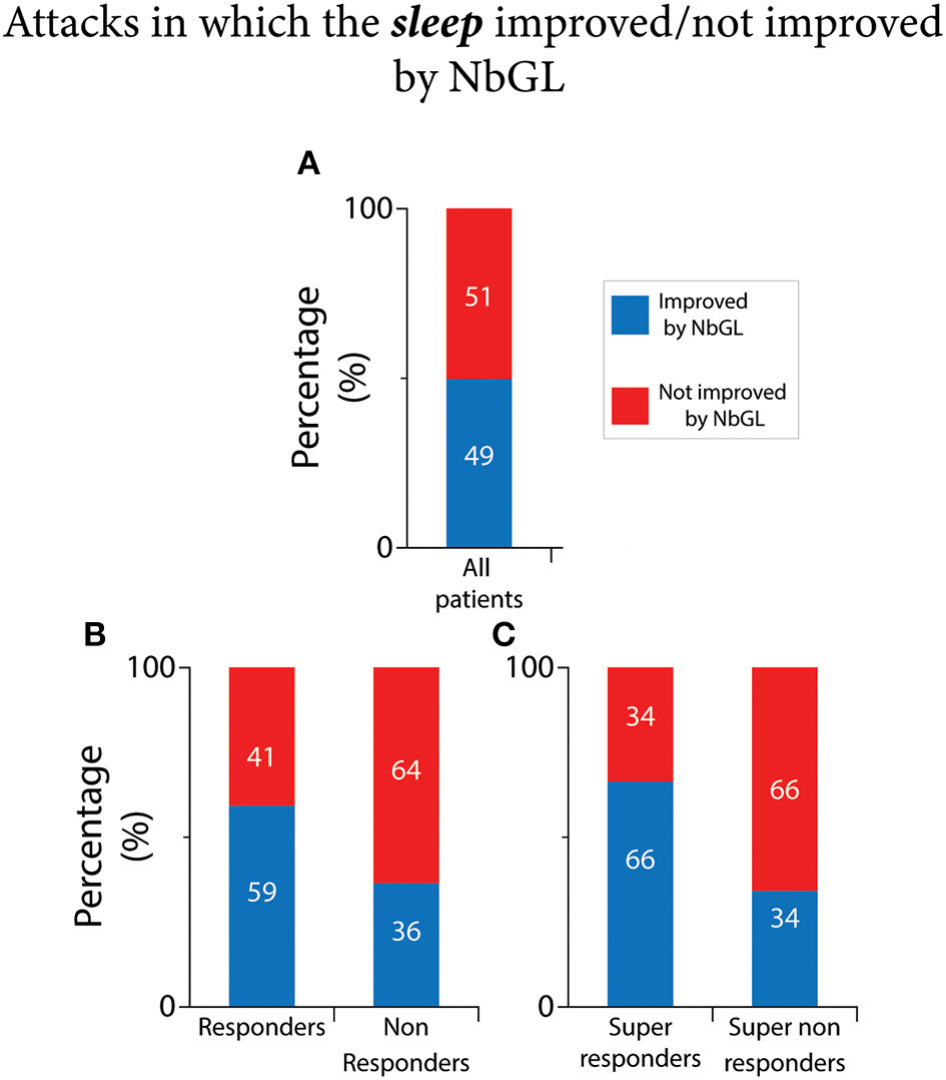
Narrow band green light (NbGL) effects on incidence of attacks in which sleep improved. **(A)** All patients. **(B)** Responders vs. non-responders. **(C)** Super-responders vs. super non-responders. The 100% (in **A–C**) represents incidences of headache improvement if all attacks in which the NbGL was used (all, 1,588/3,232; responders, 1,071/1,803; non-responders, 517/1,429; super-responders, 837/1,260; super non-responders, 353/1,035).

As shown in Figure 7 and Table 2, of the 3,232 recorded attacks, same day sleep (i.e., sleep during the night that followed the day NbGL was used) was improved in 49% (1,588 attacks) by the NbGL. In the 1,803 headache attacks recorded by the 110 responders, sleep was improved in 59% (1,071 attacks) by the exposure to NbGL. In contrast, In the 1,429 headache attacks recorded by the 71 non-responders, anxiety was improved in only 36% (517 attacks) by the exposure to NbGL (*p* < 0.0001 DF = 1, Fisher exact). In the 1,260 headache attacks recorded by the 77 super-responders, anxiety was improved in 66% (837 attacks) by the NbGL. In contrast, in the 1,035 headache attacks recorded by the 49 super non-responders, anxiety was improved in only 34% (353 attacks) by the NbGL (*p* < 0.0001 DF = 1, Fisher exact).

### Effect of treatment duration

**Figure 8:**
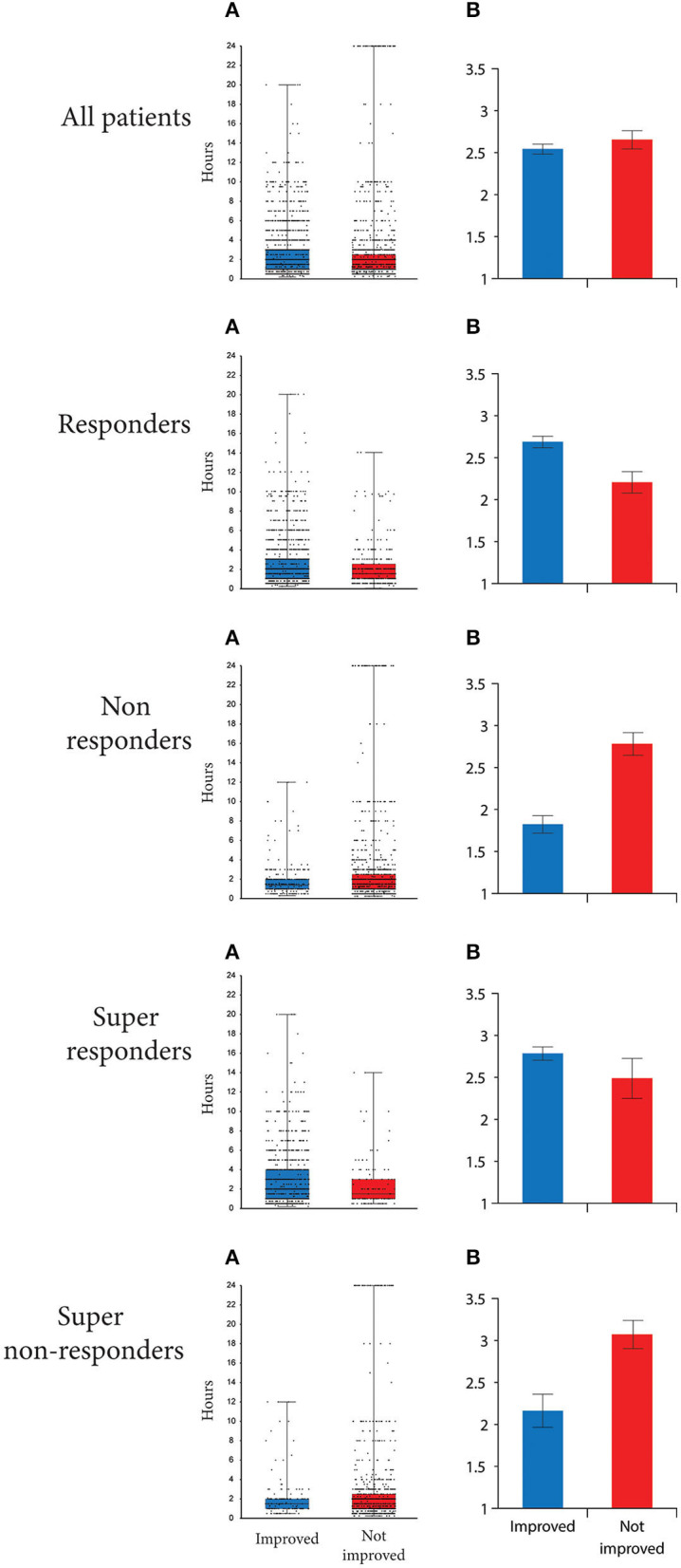
Time individual patients spent in NbGL. (A_s_) Box-and-whisker plots [median (IQR)] combined with scatterplots of individual values are illustrated for all participants, as well as those classified as responders, non-responders, super-responders and super non-responders. (B_s_) Mean (±S.D.) time spent during attacks in which the headache improved and attacks in which the headache did not improve. Note that responders spent more time in NbGL during attacks in which the headache improved than attacks in which the headache did not improve and that non-responders spent more time in NbGL during attacks that did not improve than during attacks in which the headache improved.

As shown in Figure 8, among all participants, duration of exposure to NbGL was 30 min longer in attacks in which the headache improved than in attacks in which it didn't [2 (1–3) vs. 1.5 (1–2.5) h, median (IQR), *p* < 0.001, Mann Whitney, Figure 8-Top]. Similarly, in the responders, duration of exposure to NbGL was 30 min longer in attacks in which the headache improved than in attacks in which it didn't [2 (1,2,3) vs. 1.5 (1–2.5) h, *p* < 0.001, Mann Whitney]. In contrast, in the non-responders, duration of exposure to NbGL was not longer in attacks in which the headache improved vs. attacks in which it didn't [1.4 (1,2) vs. 1.5 (1–2.5) h, *p* = 0.11 Mann Whitney]. As for the super-responders, duration of exposure to NbGL was also longer in attacks in which the headache improved vs. attacks in which it didn't [2 (1–4) vs. 1.5 (1–3) h]. Although not significantly different, a nonsignificant trend was observed (*p* = 0.083, Mann Whitney). For the super non-responders, duration of exposure to NbGL was similar in attacks in which the headache improved and attacks in which it didn't [1.5 (1,2) vs. 1.5 (1–2.5) h, median (IQR), *p* = 0.95 Mann Whitney].

## Discussion

The goal of this prospective, observational, open-label, real world study was to determine whether treatment with NbGL during the ictal phase of migraine, improves patients' perception of their headache, photophobia, anxiety and same-night sleep. Using daily diaries to capture patients' perception of headache, for ≥50% responders this goal was achieved in 61% of participants but not in the other 39%. For ≥75% responders, this goal was achieved in 42% of participants while 27% were super non-responders, responding in <25% of attacks. As for duration of exposure, it appears that a period of 2 h is sufficient as both responders and non-responders see no further benefit by using it longer. Based on these results, we conclude that by spending time in an environment in which the ambient light is a dim narrow band of green (as delivered in this study by the Allay lamp), about 60% of migraine patients may experience improvement in their headache. As the rate of improvement in photophobia, anxiety and sleep among the responders was lower than the rate of improvement in headache, we cannot rule out the possibility that these improvements were secondary to the improvement in the headache itself.

Regarding photophobia and sleep, where rates of improvement among non-responders (photophobia-35%, sleep-39% of attacks) exceeds the rate of headache improvement (21% of attacks), we cannot rule out the possibility that the observed improvements were achieved (at least) partially through NbGL interactions with pathways that are independent of those involved in the perception of headache. As for anxiety, however, where rates of improvement among responders (46% of attacks) and non-responders (18% of attacks) were lower than rates of improvement in headache (responders−82%, non-responders−21% of attacks), it is possible that the observed improvement is secondary to the improvement in the headache itself rather than a direct NBGL effect on the perception of anxiety.

Attempting to explain the NbGL effects, the Burstein group used electro-retinography and visual evoked potential recording in migraine patients demonstrating that the electrical signals the eyes send to the brain are smallest in response to NbGL than to all other colors of light, and that the electrical signals generated in the cortex in response to NbGL are significantly smaller than signals generated by blue, red, amber, and white lights (4). Shifting to animal studies, they described direct monosynaptic retinal projections to (a) trigeminovascular thalamic neurons that relay photic signals to the somatosensory, visual, auditory and olfactory cortices and (b) hypothalamic neurons involved in the regulation of sleep, mood, appetite, pain modulation, and sympathetic and parasympathetic tone (5). Based on the distributions of these functional/anatomical retinal projections, they proposed that NbGL effects on the headache itself and the sensitivity to light could be mediated mainly through the retino-thalamo-cortical projections (1, 2), that NbGL effects on sleep are mediated through the dense retinal projections to hypothalamic neurons containing orexin, histamine, and melanin-concentrating hormone (5), and that retinal projections to hypothalamic neurons that regulate sympathetic and parasympathetic functions involved in the production of physiological correlates of anxiety (e.g., perception of increased heart rate, throat tightness, light headedness) could contribute to migraine patients, perception that the NbGL reduces their anxiety (5). However, the well-established reciprocal functional connections between brain areas involved in the generation of headache, visual perception, sleep and anxiety, raise the possibility that improvement in one of these perceptions (e.g., headache) can influence the others. More global effects have been proposed to include NbGL ability to enhance endogenous opioid secretion (7, 8).

Taken into consideration the nature of this study and the absence of a placebo arm, no real comparisons could be made between the efficacy of NbGL and FDA approved abortive migraine drugs such as triptans and gepants. Nevertheless, given that the study intervention was classified as imposing less than minimal risk, and consequently did not meet criteria for requiring FDA approval, that the intervention is affordable (estimated cost of $10/year), and that 61% of the patients reported that it reduced their headache experience in >50% of the attacks, photophobia in >65% of the attacks, and migraine-associated anxiety in >50% of the attacks, the findings support the conduct of a large, multi-center, randomized controlled trial.

### Study strengths and limitations

The study included a substantial number of patients and a large number of attacks. Because participants were not recruited through clinics or by clinicians there is less potential for biases related to investigator expectations. Limitations include the absence of a contemporaneous control group and a modest participation rate among potentially eligible purchasers of the Allay Lamp. If participants who did well with treatment were more likely to participate that could limit the generalizability of these results to all Allay users. Since individuals purchased the Lamp, their expectations may have inflated efficacy. Finally, though we report diary based outcomes, preventive studies often include a broader set of outcome measures. Thus, while results support the use of this non-invasive, safe and affordable complementary approach, they also justify the conduct of controlled studies with a broader set of outcome measures.

## Data availability statement

The raw data supporting the conclusions of this article will be made available by the authors, without undue reservation.

## Ethics statement

The studies involving humans were approved by Ethical and Independent Review Services (Independence, MO, USA). The studies were conducted in accordance with the local legislation and institutional requirements. The participants provided their written informed consent to participate in this study.

## Author contributions

RL: Conceptualization, Formal analysis, Methodology, Writing—original draft, Writing—review and editing. AM-C: Data curation, Formal analysis, Writing—original draft, Writing—review and editing. MS: Project administration, Software, Writing—review and editing. MR: Conceptualization, Data curation, Formal analysis, Investigation, Methodology, Project administration, Software, Supervision, Writing—review and editing. SA: Writing—review and editing. TH: Data curation, Formal analysis, Methodology, Writing—original draft, Writing—review and editing. RB: Conceptualization, Resources, Supervision, Writing—original draft, Writing—review and editing.
